# Invasive Pulmonary Mucormycosis Due to Rhizopus oryzae in a Patient With Uncontrolled Diabetes

**DOI:** 10.7759/cureus.110834

**Published:** 2026-06-14

**Authors:** Henry Quin, Amit Sharma, Anil Kumar Singh, Rohit Sharma

**Affiliations:** 1 Internal Medicine, Mass General Brigham Salem Hospital, Salem, USA; 2 Infectious Disease, Geisinger Health System, Scranton, USA; 3 Internal Medicine, Geisinger Health System, Scranton, USA

**Keywords:** cavitary lesion, diabetes mellitus, invasive fungal infection, pulmonary mucormycosis, rhizopus oryzae

## Abstract

A man in his 60s with poorly controlled type 2 diabetes mellitus presented with subacute shortness of breath and hemoptysis. Imaging demonstrated extensive bilobar cavitary destruction of the left lung with pleural extension. Bronchoalveolar lavage cultures isolated Rhizopus oryzae, and intravenous liposomal amphotericin B was commenced. Definitive surgical management via pneumonectomy was precluded by the patient's severe cardiomyopathy and limited pulmonary reserve. The patient was discharged on prolonged antifungal therapy but was readmitted within one week with recurrent hemoptysis. Bronchial artery embolization was deemed too high risk. He subsequently developed catastrophic hemorrhage and died. This case highlights the rapid clinical progression of diabetic pulmonary mucormycosis and underscores the poor prognosis when patient comorbidities preclude surgical source control.

## Introduction

Mucormycosis is a rare but severe invasive fungal infection caused by moulds of the order Mucorales [1,2]. It predominantly affects individuals with immunocompromising conditions, including hematological malignancy, solid organ or hematopoietic stem cell transplantation, and poorly controlled diabetes mellitus [1,2]. Pulmonary mucormycosis represents a highly lethal manifestation of this disease, presenting significant diagnostic challenges due to its clinical and radiological overlap with tuberculosis, necrotizing pneumonia, and bacterial lung abscesses [3]. Furthermore, diagnostic efforts are often hampered by the limited sensitivity of conventional serum fungal biomarkers, such as β-D-glucan and galactomannan, which frequently yield false-negative results in patients with mucormycosis [1,4].

Other clinical forms include rhino-orbito-cerebral, gastrointestinal, cutaneous, renal, and isolated central nervous system disease, all of which are associated with substantial morbidity and mortality if diagnosis and treatment are delayed [1]. Liposomal amphotericin B remains the cornerstone of therapy, with posaconazole or isavuconazole commonly used as step-down or salvage treatment [1,5].

This report describes aggressive pulmonary mucormycosis presenting as extensive cavitary lung disease in a patient with uncontrolled diabetes, in the absence of other classical immunocompromising conditions.

## Case presentation

A man in his 60s with type 2 diabetes mellitus, cardiomyopathy with reduced ejection fraction (40%), bilateral carotid artery disease, peripheral vascular disease, hypertension, hyperlipidaemia, diverticulosis, and gastro-oesophageal reflux disease presented with two weeks of progressive shortness of breath and hemoptysis. He denied recent travel, alcohol misuse, or illicit drug use, and had a 40-year smoking history, recently quitting. Surgical history included prior shoulder arthroscopy for rotator cuff repair. 

On presentation, vital signs were stable: BP 118/58 mmHg, HR 83 bpm, temperature 37 °C, RR 18/min, oxygen saturation 96% on room air. Physical exam was notable for cachexia.

Laboratory evaluation demonstrated poorly controlled diabetes and mild renal impairment: HbA1c 14.7%, serum creatinine 1.4 mg/dL (eGFR 56 mL/min/1.73 m²), BUN 33 mg/dL, sodium 133 mmol/L, albumin 3.1 g/dL, and normocytic anaemia (Hb 9.4 g/dL) with normal WCC and differential. A comprehensive summary of the patient's laboratory findings on admission is provided in Table 1.

**Table 1:** Laboratory findings on initial presentation

Laboratory Parameter	Patient Value	Reference Range	Units
Glycated Hemoglobin (HbA1c)	14.7	< 5.7	%
Microbiology & Biomarkers			
Bronchoalveolar Lavage (BAL) Culture	Positive for Rhizopus oryzae	Negative	—
Serum (1,3)-Beta-D-glucan	Negative (<60)	< 60	pg/mL
Serum Galactomannan Antigen	Negative (<0.5)	< 0.5	Index
Hematology (CBC)			
White Blood Cell (WBC) Count	8.53	4.5 – 11.0	× 10³/µL
Red Blood Cell (RBC) Count	3.2	4.50 – 5.90	× 10⁶/µL
Hemoglobin	9.4	13.8 – 17.2	g/dL
Hematocrit	30	41.0 – 50.0	%
Mean Corpuscular Volume (MCV)	93.8	80.0 – 100.0	fL
Mean Corpuscular Hemoglobin (MCH)	29.4	27.0 – 33.0	pg
MCHC	31.3	32.0 – 36.0	g/dL
Red Cell Distribution Width (RDW)	15.6	11.5 – 14.5	%
Platelet Count	160	150 – 450	× 103/muL
Mean Platelet Volume (MPV)	10.6	9.4 – 12.4	fL
WBC Differential			
Neutrophils	57	40.0 – 70.0	%
Lymphocytes	30	20.0 – 44.0	%
Monocytes	11	2.0 – 10.0	%
Eosinophils	1.2	0.0 – 5.0	%
Basophils	0.2	0.0 – 2.0	%
Immature Granulocytes	0.6	0.0 – 1.0	%
Comprehensive Metabolic Panel (CMP)			
Serum Glucose	101	70 – 100	mg/dL
Blood Urea Nitrogen (BUN)	33	7 – 20	mg/dL
Serum Creatinine	1.4	0.7 – 1.3	mg/dL
eGFR	56	≥ 90	mL/min/1.73m²
Serum Sodium	133	135 – 145	mmol/L
Serum Chloride	95	98 – 107	mmol/L
Carbon Dioxide (CO2)	25	22 – 29	mmol/L
Anion Gap	13	3 – 11	mmol/L
Albumin	3.1	3.5 – 5.0	g/dL
Total Protein	6.2	6.4 – 8.3	g/dL
Aspartate Aminotransferase (AST)	20	10 – 40	U/L
Alkaline Phosphatase	105	44 – 147	U/L
Total Bilirubin	0.3	0.2 – 1.2	mg/dL
Serum Calcium	8.6	8.6 – 10.3	mg/dL
Serum Magnesium	2.5	1.7 – 2.2	mg/dL

**Figure 1:**
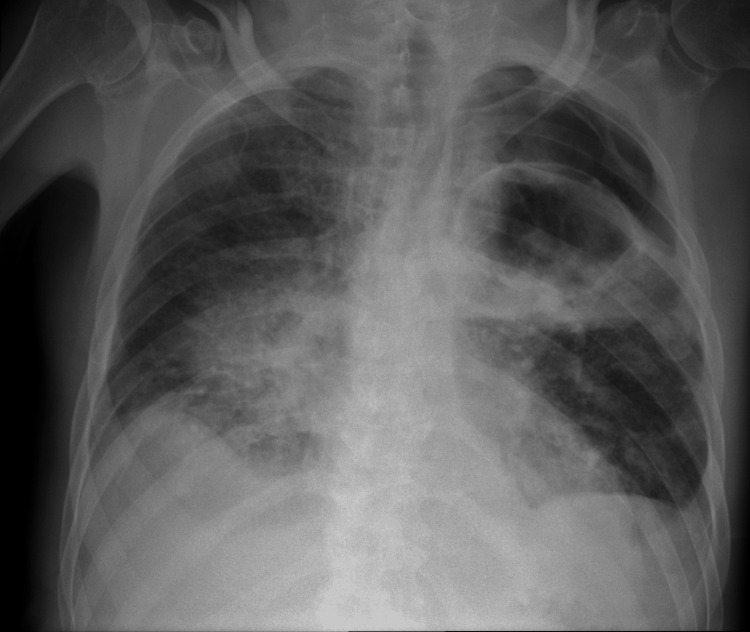
Initial chest X-ray demonstrating multifocal pulmonary involvement There are prominent right perihilar airspace opacities. Notably, a large, thick-walled cavitary lesion is visualized in the left lung apex.

**Figure 2:**
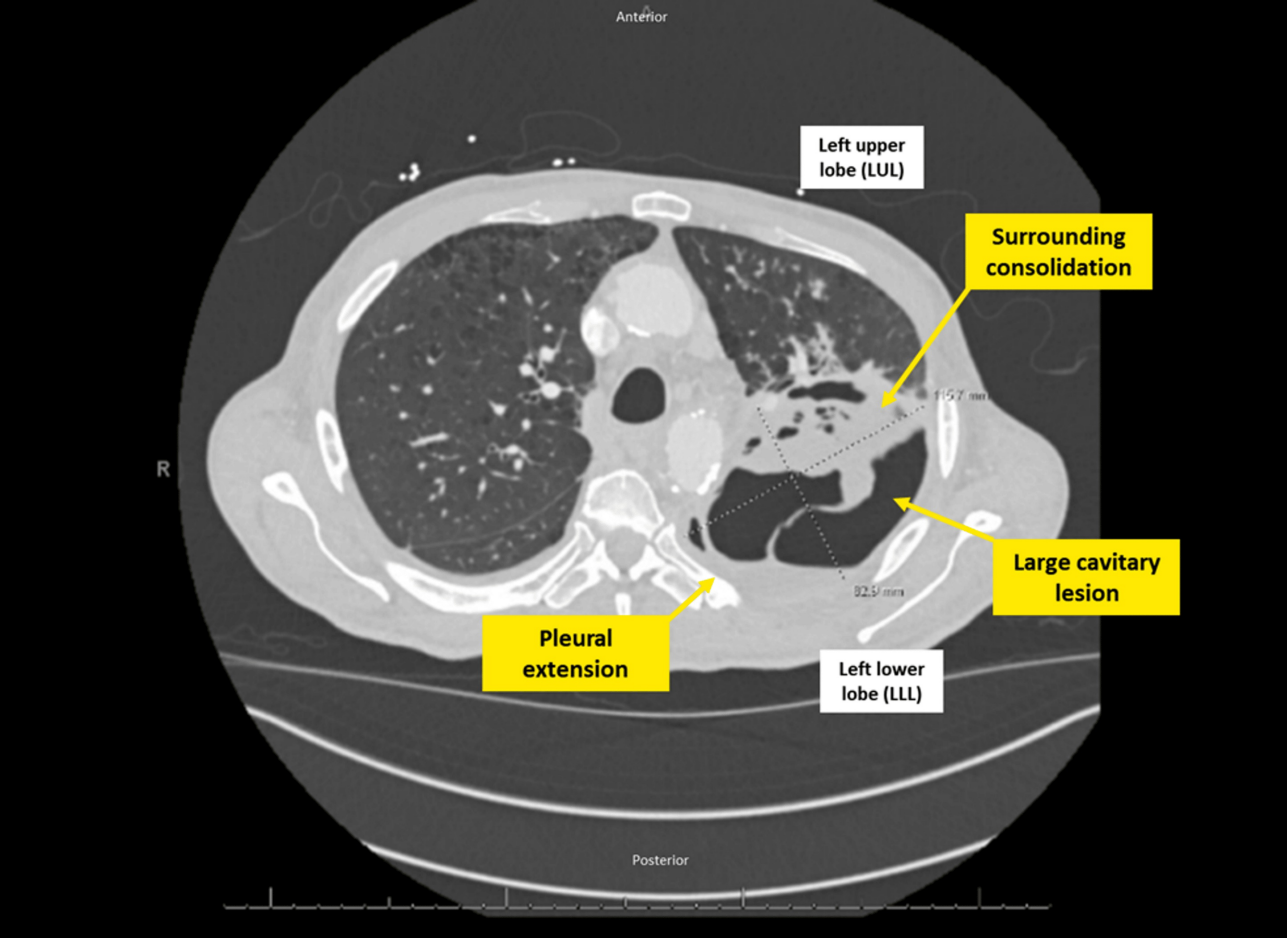
Computed Tomography (CT) of the chest revealing a large, complex necrotic cavity The lesion involves the apicoposterior segment of the left upper lobe and extends into the superior segment of the left lower lobe. There is evidence of direct erosion into the pleural space.

Chest radiography revealed right perihilar airspace opacities and a large left apical cavitary lesion (Figure 1). CT chest demonstrated extensive bilobar cavitary disease involving the left upper and lower lobes, with pleural extension and chest wall erosion. New peribronchial consolidations in the anterior left upper lobe suggested airway spread (Figure 2).

The initial differential included bacterial lung abscess, necrotizing pneumonia, pulmonary tuberculosis, and invasive fungal pneumonia. The presence of extensive cavitation with pleural extension and progressive hemoptysis suggested an angioinvasive process concerning for invasive fungal disease. Serum β-D-glucan and galactomannan assays were negative.

Flexible bronchoscopy with bronchoalveolar lavage grew molds consistent with Mucorales; final speciation confirmed Rhizopus oryzae. Intravenous liposomal amphotericin B (5 mg/kg/day) was initiated promptly. Additional empiric voriconazole was initially administered until definitive identification confirmed Mucorales, after which it was discontinued.

Thoracic surgical assessment determined that tissue biopsy for histopathological confirmation carried an excessively high risk of complications. Furthermore, given the patient’s reduced ejection fraction and poor pulmonary reserve, surgical resection (pneumonectomy) for source control was deemed prohibitively high-risk. Consequently, medical management alone was pursued.

The patient was discharged with plans for prolonged antifungal therapy but was readmitted within one week with recurrent hemoptysis. Interventional radiology identified multiple bleeding vessels; however, bronchial artery embolization was deferred, as the underlying angioinvasive process presented a high risk of technical failure and extensive tissue infarction in the absence of surgical source control. The patient subsequently developed massive hemoptysis leading to cardiac arrest and death.

## Discussion

This case illustrates a rapidly fatal course of pulmonary mucormycosis in a patient with uncontrolled diabetes mellitus, in the absence of other classical immunocompromising conditions. Diabetes is a major global risk factor for mucormycosis and accounts for a substantial proportion of cases worldwide [1]. Poor glycemic control compromises innate immunity, particularly neutrophil chemotaxis and phagocytosis, and increases free serum iron, thereby promoting the growth and angioinvasion of Rhizopus species. These organisms further enhance iron acquisition by producing siderophores, which are iron-chelating molecules, allowing them to thrive in the acidic, hyperglycemic environment characteristic of poorly controlled diabetes [6,7].

The hallmark of this infection is its angioinvasive process, which leads to vascular thrombosis, tissue infarction, and widespread necrosis. This vascular compromise frequently manifests clinically as recurrent hemoptysis and can culminate in catastrophic hemorrhage if the infection breaches larger pulmonary vessels such as the pulmonary arteries [8]. These pathophysiological processes are mirrored radiologically by the extensive bilobar cavitary disease and chest wall erosion observed in this patient.

Pulmonary mucormycosis is among the most lethal clinical manifestations of invasive mucormycosis, with a pooled mortality of 57%, though outcomes have improved over time [1,2]. Clinical presentation is often non-specific, with symptoms including cough, dyspnoea, fever, and hemoptysis. Radiographic features, such as extensive cavitary destruction with pleural extension and chest wall erosion, are suggestive of invasive fungal disease, but can overlap with bacterial lung abscess, necrotizing pneumonia, and pulmonary tuberculosis, frequently resulting in diagnostic delay [4].

Definitive diagnosis requires histopathological demonstration of broad, pauciseptate hyphae with right-angle branching and confirmation by culture. Conventional fungal biomarkers, including serum β-D-glucan and galactomannan, are typically negative in mucormycosis because Mucorales lack these cell wall components, which may falsely reassure clinicians and delay initiation of appropriate therapy [4]. Molecular diagnostic techniques, including PCR assays targeting Mucorales DNA, demonstrate overall sensitivity of 85.7% and specificity of 94.7%, though sensitivity varies substantially by specimen type, with lower sensitivity in blood compared with bronchoalveolar lavage fluid [9]. These assays are not yet standardized for routine clinical use, and the statistical limitations reinforce the importance of maintaining clinical suspicion and pursuing invasive diagnostic sampling when mucormycosis is suspected.

Liposomal amphotericin B at a dose of 5 mg/kg/day remains first-line therapy and should be initiated promptly when mucormycosis is suspected or confirmed [1,6]. Delays in amphotericin B-based therapy are strongly associated with increased mortality [10]. Step-down or salvage therapy with posaconazole or isavuconazole may be effective and better tolerated for prolonged treatment courses [6]. Voriconazole is inactive against Mucorales but is frequently initiated empirically to cover Aspergillus species until definitive microbiological identification is available.

Surgical resection is a critical adjunct to antifungal therapy and is independently associated with improved survival. Meta-analytic data demonstrate a 32% absolute risk reduction in mortality with combined medical-surgical management compared with medical therapy alone, with median survival of 406 days versus 28 days, respectively [2,5]. Surgery reduces fungal burden and removes necrotic tissue, which otherwise limits antifungal penetration. However, extensive pulmonary involvement or prohibitive cardiopulmonary comorbidities may preclude operative intervention. In this patient, definitive management would have required pneumonectomy, which was contraindicated due to severely reduced ejection fraction and limited pulmonary reserve. The inability to achieve surgical source control, combined with advanced angioinvasive disease, likely contributed to the fatal outcome.

Current international guidelines recommend early initiation of high-dose liposomal amphotericin B combined with early and aggressive surgical debridement, after correction of metabolic derangements, whenever feasible [1]. When surgery is not possible, prognosis remains poor despite optimal medical therapy. This case underscores that in pulmonary mucormycosis, surgical resection remains a key determinant of survival. When surgery is contraindicated by severe comorbidities, as in this patient, outcomes remain poor despite optimal antifungal therapy, highlighting the critical importance of early multidisciplinary evaluation and the need for novel therapeutic strategies.

## Conclusions

Pulmonary mucormycosis is a highly lethal infection that must be considered in patients with uncontrolled diabetes, even in the absence of traditional immunocompromising factors. Because standard fungal biomarkers (β-D-glucan and galactomannan) are typically negative, diagnosis relies on a high index of clinical suspicion and early invasive sampling.

Survival is fundamentally dependent on a combined-modality approach: the immediate initiation of liposomal amphotericin B and early surgical resection. As this case illustrates, when comorbidities preclude surgical debridement, outcomes remain poor despite optimal medical therapy. This underscores the critical importance of early multidisciplinary evaluation and the need for prompt medical and surgical intervention to improve survival.
